# Fabrication of carbon nanospheres by the pyrolysis of polyacrylonitrile–poly(methyl methacrylate) core–shell composite nanoparticles

**DOI:** 10.3762/bjnano.8.190

**Published:** 2017-09-11

**Authors:** Dafu Wei, Youwei Zhang, Jinping Fu

**Affiliations:** 1 Key Laboratory for Ultrafine Materials of Ministry of Education, School of Materials Science and Engineering, East China University of Science and Technology, Shanghai 200237, China,; 2 State Key Laboratory for Modification of Chemical Fibers and Polymer Materials, College of Materials Science and Engineering, Donghua University, Shanghai 201620, China

**Keywords:** carbon nanospheres, core–shell nanoparticles, emulsion polymerization, polyacrylonitrile

## Abstract

Carbon nanospheres with a high Brunauer–Emmett–Teller (BET) specific surface area were fabricated via the pyrolysis of polyacrylonitrile–poly(methyl methacrylate) (PAN–PMMA) core–shell nanoparticles. Firstly, PAN–PMMA nanoparticles at high concentration and low surfactant content were controllably synthesized by a two-stage azobisisobutyronitrile (AIBN)-initiated semicontinuous emulsion polymerization. The carbon nanospheres were obtained after the PAN core domain was converted into carbon and the PMMA shell was sacrificed via the subsequent heat treatment steps. The thickness of the PMMA shell can be easily adjusted by changing the feeding volume ratio (FVR) of methyl methacrylate (MMA) to acrylonitrile (AN). At an FVR of 1.6, the coarse PAN cores were completely buried in the PMMA shells, and the surface of the obtained PAN–PMMA nanoparticles became smooth. The thick PMMA shell can inhibit the adhesion between carbon nanospheres caused by cyclization reactions during heat treatment. The carbon nanospheres with a diameter of 35–65 nm and a high BET specific surface area of 612.8 m^2^/g were obtained from the PAN–PMMA nanoparticles synthesized at an FVR of 1.6. The carbon nanospheres exhibited a large adsorption capacity of 190.0 mg/g for methylene blue, thus making them excellent adsorbents for the removal of organic pollutants from water.

## Introduction

Due to their high specific surface area, chemical inertness, good mechanical stability and unique electrical properties, carbon nanospheres have numerous potential applications in nanocomposites [1], gas storage [2], lithium batteries [3–5], fuel cells [6–7], supercapacitors [8–9], catalysis carriers [10–11], drug delivery [12–13] and adsorption [14–15]. Various techniques, including arc discharge [16], laser ablation [17], chemical vapor deposition [18], and solvothermal method [19], have been developed for the production of carbon nanospheres. However, these methods require special equipment, and the size control of the resulting spheres also remains a problem.

Carbon nanospheres or microspheres can also be fabricated via pyrolysis of various particulate polymer precursors [20–27]. The size and morphology of the resulting carbon nanospheres are closely related to those of the precursor. Thus, the pyrolysis method can produce carbon nanospheres with well-controlled size and morphology by adjusting the size and morphology of the precursor.

As is well known, after preoxidization and carbonization treatments, polyacrylonitrile (PAN) can be converted into carbon. High-performance carbon fibers, carbon nanofiber membranes, 3D-ordered carbon materials, and carbon nanoparticles have been fabricated from various PAN precursors [28–35]. Discrete and well-defined carbon nanospheres were first fabricated by Kowaleski et al. [31] via successive shell crosslinking, oxidization and carbonization of the micelles resulting from the self-assembly of PAN-*b*-poly(acrylic acid) (PAA) block copolymer in water. The crosslinked shell can retain the micelle structure during the heat treatments, and thus is very crucial to obtain discrete carbon nanospheres after pyrolysis. Jérom et al. [32] obtained discrete carbon nanocapules after pyrolysis of stable PAN-*b*-PAA micelles with Au-nanoparticle-crosslinked cores. However, the difficulty in synthesis of the block copolymers and the low efficiency of micellization make the method unappealing to the industrial community.

Emulsion polymerization is a facile and efficient route to synthesize polymer particles. By combining the emulsion polymerization with the pyrolysis, the production efficiency of PAN-based carbon nanospheres can be improved substantially, which is very important to industrial manufacturing. Yang et al. obtained carbon nanospheres with a diameter of 170–190 nm from PAN particles synthesized by a soap-less emulsion polymerization method [35].

A series of complicated chemical reactions, including oxidization, dehydrogenation, cyclization, and so on, occur during the heat treatment of PAN. As the size of the PAN nanoparticles decreased to less than 100 nm, the aggregations between PAN nanoparticles as well as the inter-particular crosslinking via cyclization reactions exacerbate during the heat treatment, resulting in serious adhesion between the carbon nanoparticles. As a result, an agglomerated carbon bulk instead of discrete carbon nanoparticles was obtained [33–34]. To solve this problem, Wu et al. [33] coated a protective layer of inorganic salt, titanium phosphate (TP), on the surfaces of the PAN nanoparticles. The adhesion was inhibited, and a few discrete carbon nanoparticles were obtained. However, the carbonized product still contained many agglomerates of carbon nanoparticles with partial merged edges. The possible reason is that the coated TP layer was too thin to completely cover the surface of the PAN nanoparticles. Furthermore, an extra acid-washing process was needed to remove the inorganic salt layer [33,36].

As the cyclization reactions between nitriles mainly occur during the preoxidization and low-temperature carbonization steps (<450 °C), we tried to coat with a protective poly(methyl methacrylate) (PMMA) layer, which would remain stable during the preoxidation and the low-temperature carbonization. This layer, however, will completely degrade after the high-temperature carbonization on the surface of the PAN nanoparticle to inhibit the inter-particular adhesion between carbon nanospheres; thus, the direct fabrication of discrete carbon nanospheres can be achieved.

Although carbon nanospheres and microspheres have been fabricated by various methods, reports on the fabrication of monodisperse carbon nanospheres with a diameter less than 100 nm are still very few [21,27,37–38]. Also, although PAN-based carbon nanospheres and microspheres were reported to be fabricated by pyrolysis of various precursors [23,31,33–35], their detailed microstructure, including the elemental composition, crystalline structure, structure ordering and porous configuration, have not yet been reported.

**Figure 1:**
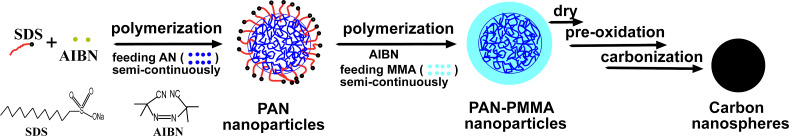
Schematic of the fabrication of PAN-based carbon nanospheres.

In this study, PAN-based carbon nanopsheres were fabricated from the PAN–PMMA core–shell nanoparticles precursor. Specifically, PAN–PMMA core–shell latexes at high concentration and low surfactant content were first synthesized via a two-stage AIBN-initiated semicontinuous emulsion polymerization. The PAN–PMMA latex was converted into carbon nanospheres after successive drying and heat treatments (Figure 1). The composition, structure and morphology of the PAN–PMMA nanoparticles and the carbon nanospheres were characterized in detail. The obtained carbon nanospheres with a diameter of 35–65 nm exhibit a high BET specific surface area of 612.8 m^2^/g and a large adsorption capacity of 190.0 mg/g for methylene blue, thus making them excellent adsorbents for the removal of organic pollutants from water.

## Experimental

### Materials

Acrylonitrile (AN) was distilled before use to remove the inhibitor. Methyl methacrylate (MMA) was distilled at a reduced pressure before use to remove the inhibitor. Sodium dodecyl sulphate (SDS) and methylene blue (MB) were used without further puriﬁcation. 2,2'-azobisisobutyronitrile (AIBN) was used as the initiator and purified via recrystallization in methanol. Deionized water was used in all the synthesis processes.

### Preparation of PAN–PMMA latex

#### Preparation of seed PAN nanolatex

The seed PAN latex nanomaterial (nanolatex) was prepared via an AIBN-initiated semicontinuous emulsion polymerization method [39]. The typical process was as follows. 150 mg of AIBN, 400 mg of SDS and 120 mL of deionized water were placed into a flask, and this was followed by purging with nitrogen under stirring for 20 min to remove oxygen. The solution was then heated to 67 °C and maintained at that temperature. 15 mL of AN was fed continuously at a rate of 15 mL/h. After the AN feed step, the reaction was continued for 4 h under nitrogen protection and constant stirring. Finally, the PAN nanolatex was cooled, and the unreacted AN was removed at a reduced pressure.

#### Preparation of PAN–PMMA latex

The typical process was as follows. First, 80 mg of AIBN and 60 mL of the PAN nanolatex were placed into a flask, and this was followed by purging with nitrogen under stirring for 30 min to remove oxygen. Then, the dispersion was heated to 70 °C and maintained at that temperature. 6 mL of MMA was fed continuously at a rate of 4 mL/h. After completion of the MMA feed step, the reaction was continued for 3.5 h under nitrogen protection and constant stirring. Finally, the PMMA–PAN latex was cooled and the unreacted MMA was removed at a reduced pressure.

Varying the feeding volume ratio (FVR) of MMA to AN, three PAN–PMMA latexes were prepared. The PAN–PMMA latexes prepared at an FVR of 0.8, 1.6, and 2.4 are denoted as PAN-PMMA1, PAN-PMMA2, and PAN-PMMA3, respectively. All the synthesis experiments were repeated at least once to ensure reliability.

#### Preparation of PAN-based carbon nanospheres

PAN-based carbon nanospheres were fabricated via successive drying, preoxidation and carbonization steps of the PAN–PMMA latex. First, the PAN–PMMA latex was freeze-dried into a powder. Then, the powder was preoxidized in an air circulating oven, which was heated to 250 °C at a rate of 4 °C/min and remained for 4 h, and then cooled down naturally. Finally, the preoxidized product was carbonized in a high-temperature carbonization furnace, which was filled with pure nitrogen and heated to a given temperature at a rate of 5 °C/min and remained for 1 h (600 °C and 750 °C) or 0.5 h (1000 °C). After naturally cooling down to room temperature, the PAN-based carbon nanospheres were obtained.

### Adsorption experiment

The obtained carbon nanospheres were used as the adsorbent for the removal of methylene blue (MB) from water. A batch adsorption experiment was operated by mixing the carbon nanospheres (0.010 g) and MB aqueous solution (30 mL, 66 mg/L) in a 50 mL centrifuge tube, which was followed by shaking the mixture at a speed of 150 rpm. After 2 h of shaking, the adsorbent was separated from the mixture by centrifugation. The appearance of the mixture remained homogeneous during the adsorption experiment. The MB concentration of the supernatant was determined with a UV–vis spectrophotometer at a wavelength of 664 nm (Supporting Information File 1, Figure S1, Figure S2, Table S1). The adsorption capacity and removal efficiency of MB for carbon nanospheres were obtained via the following equations.

[1]
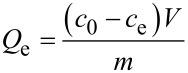


[2]
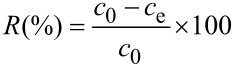


where *Q*_e_ (mg/g) is the adsorption capacity, *c*_0_ (mg/L) and *c*_e_ (mg/L) are the initial and equilibrium concentrations of MB, respectively, *V* (L) is the volume of MB aqueous solution, and *m* (g) is the weight of carbon nanospheres.

### Characterization

The z-average hydrodynamic diameter of the various nanoparticles was determined by dynamic light scattering (DLS) using a Malvern Zeta-sizer (DTS1060, Malvern, UK). The measurements were repeated three times and the average values are given. The FTIR spectra were obtained using a Nicolet Magna 550 spectrometer and KBr pellets. The Raman spectra were obtained from a Jobin Yvon T64000 Raman system. The excitation was at 514.5 nm. The laser power was set to 35 mW. The C, H and N element contents of various samples were determined using an Elementar Vario EL III element analysis system. The morphology of the various polymer nanoparticles and carbon nanospheres was observed by transmission electron microscopy (TEM) and field emission scanning electron microscopy (FESEM). The carbon nanospheres were ultrasonically dispersed in acetone. The sample for TEM observation was prepared by placing a 5 μL of particle dispersion on a copper grid, which was coated with thin films of Formvar and carbon, and was allowed to dry in air. The polymer nanoparticles were stained by mixing an equal volume of the nanoparticle dispersion and phosphate tungsic acid (PTA) aqueous solution (5 wt %) for 1 day. The PAN-PMMA1 nanoparticles were also treated by 50% NaSCN aqueous solution, a selective solvent for PAN. 75 μL of PAN-PMMA1 nanoparticle aqueous dispersion was added into 10 mL of 50% NaSCN aqueous solution under stirring. After about 20 h of stirring, the mixture was then used for the preparation of samples for TEM observation. The dried copper containing the above NaSCN-treated nanoparticles was further treated with gentle water washing to remove NaSCN salt and negative staining with the PTA aqueous solution. TEM observations were performed on a Philips CM 120 electron microscope at an accelerating voltage of 80 kV. The sample for FESEM observation was prepared by placing a drop of properly diluted nanoparticle dispersion on a clean glass sheet, and was allowed to dry in the air. FESEM observations were performed on a Hitachi S-4800 field emission scanning electron microscope. Powder X-ray diffraction (XRD) patterns were recorded using an X-ray diffractometer (Model D8 Advance, Bruker AXS) with Cu Kα radiation. Low-temperature nitrogen adsorption experiments were performed at the boiling point of nitrogen using a surface area and porosity analyzer (ASAP2020, Micromeritics). The specific surface area of the samples was calculated according to BET theory. The pore size of carbon nanospheres was calculated from the desorption branch of nitrogen isotherms using the Barret–Joyner–Halenda (BJH) method. UV–vis absorption spectra were performed on a Perkin Elmer Lambda 25 spectrophotometer.

## Results and Discussion

### PAN–PMMA nanoparticles

To realize the efficient and controlled fabrication of PAN-based carbon nanospheres from a PAN–PMMA nanoparticle precursor via the pyrolysis method, one should first realize the controlled and efficient fabrication of PAN nanoparticles and PAN–PMMA nanoparticles. Very recently, we reported a novel AIBN-initiated semicontinuous emulsion polymerization method for the synthesis of narrow-size-distribution PAN nanolatex at high AN concentration and low surfactant content [39]. Here, a PAN nanolatex with an average diameter <*D*_h_> of 96 nm and a polydispersity index of 0.03 at an AN concentration of 100 g/L (based on the volume of water) and an SDS content of 3.3% (based on the weight of monomers) was firstly fabricated by the novel emulsion polymerization method, and then used as the seed for the preparation of PAN–PMMA latex. The fabrication efficiency of the PAN–PMMA nanoparticles was thus improved substantially. Second, during the preparation of the PAN–PMMA latex, the hydrophobic initiator AIBN and semicontinuous feeding of MMA monomer were adopted to inhibit the production of secondary PMMA nanoparticles – thus realizing the selective growth of the PMMA layer on the PAN seed. Hu et al. [40] reported the synthesis of PAN–PMMA latex at a total monomer concentration of 138 g/L and a surfactant content of 10.9% by a differential microemulsion polymerization method. As compared with the result of Hu, using our two-stage AIBN-initiated emulsion polymerization method, PAN–PMMA latex can be fabricated at a higher monomer concentration (194–383 g/L) and lower surfactant content (0.87–1.71%). This is beneficial to the practical production of this materials.

### Particle size and composition analysis

**Figure 2:**
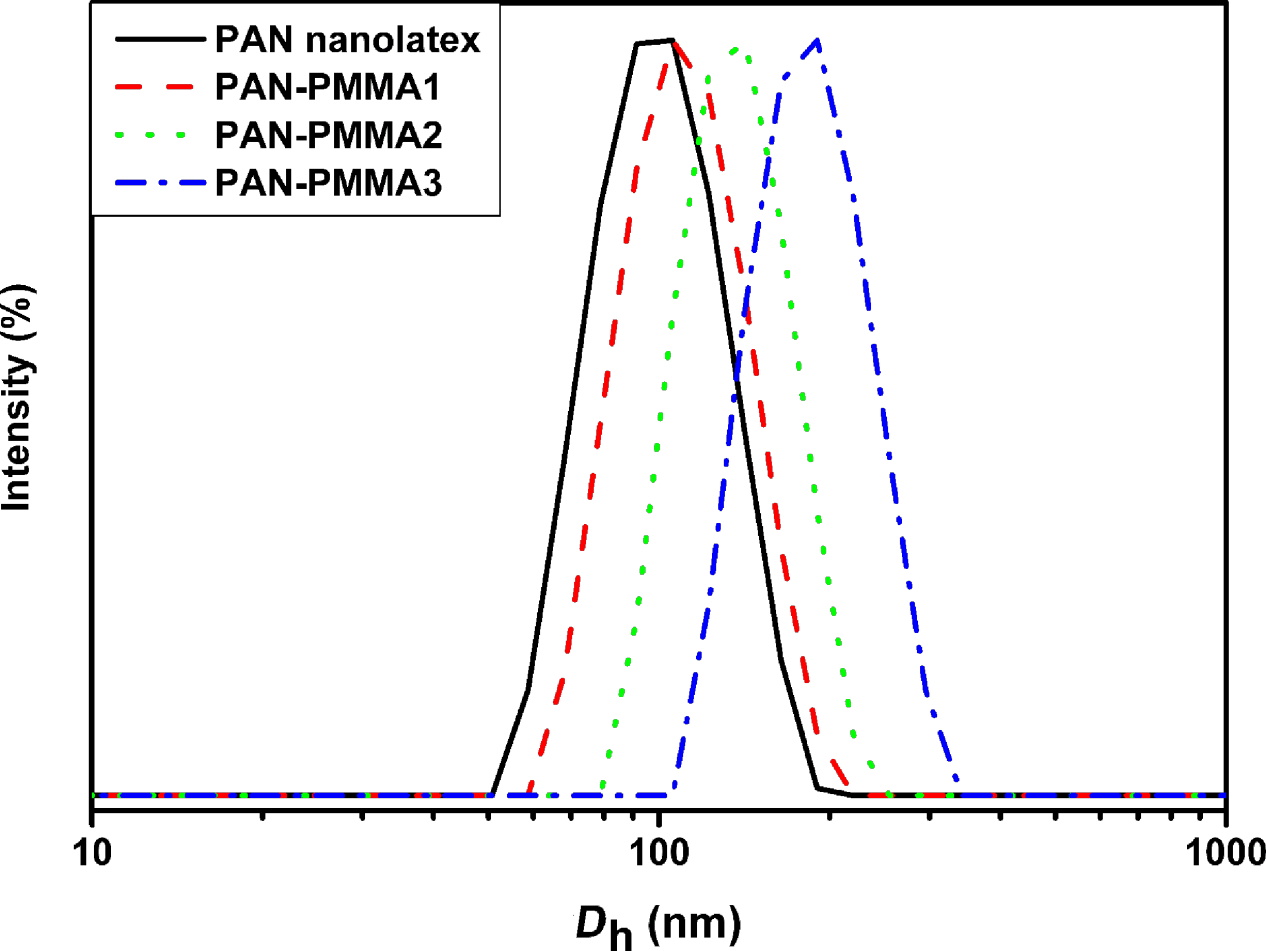
Normalized hydrodynamic diameter distribution curves of PAN nanolatex and three PAN–PMMA latexes.

The normalized hydrodynamic diameter distribution curves of seed PAN nanolatex and three PAN–PMMA latexes synthesized at different feeding volume ratios (FVR) of MMA to AN are displayed in Figure 2. The size distributions of all the latexes were fairly narrow with a PDI ranging from 0.01 to 0.06. As compared with the seed PAN nanolatex, the distribution curves of the three PAN–PMMA latexes all shift to larger diameters, in general. Also, the larger the FVR, the more the distribution curve shift to larger diameters. This indicates the formation of a PMMA outer layer on the PAN seed, as well as the fact that the thickness of the PMMA outer layer can be adjusted via simply varying the feeding monomer ratio. Furthermore, the undesirable secondary PMMA nanoparticles were not detected by DLS as indicated by the unimodal size distribution curves of the three PAN–PMMA latexes.

The z-average hydrodynamic diameter of the seed PAN nanolatex and the three PAN–PMMA latexes PAN-PMMA1, PAN-PMMA2 and PAN-PMMA3 was 96 nm, 122 nm, 154 nm and 179 nm, respectively. The weight ratio (WR) of PMMA to PAN of the PAN–PMMA nanoparticles was estimated from the N content of the PAN seed nanoparticles and the PAN–PMMA nanoparticles, determined by elemental analysis. The WR value of PAN-PMMA1, PAN-PMMA2, and PAN-PMMA3 was 1.136, 2.238 and 3.179, respectively (See Supporting Information File 1, Table S2 ).

### FTIR analysis

**Figure 3:**
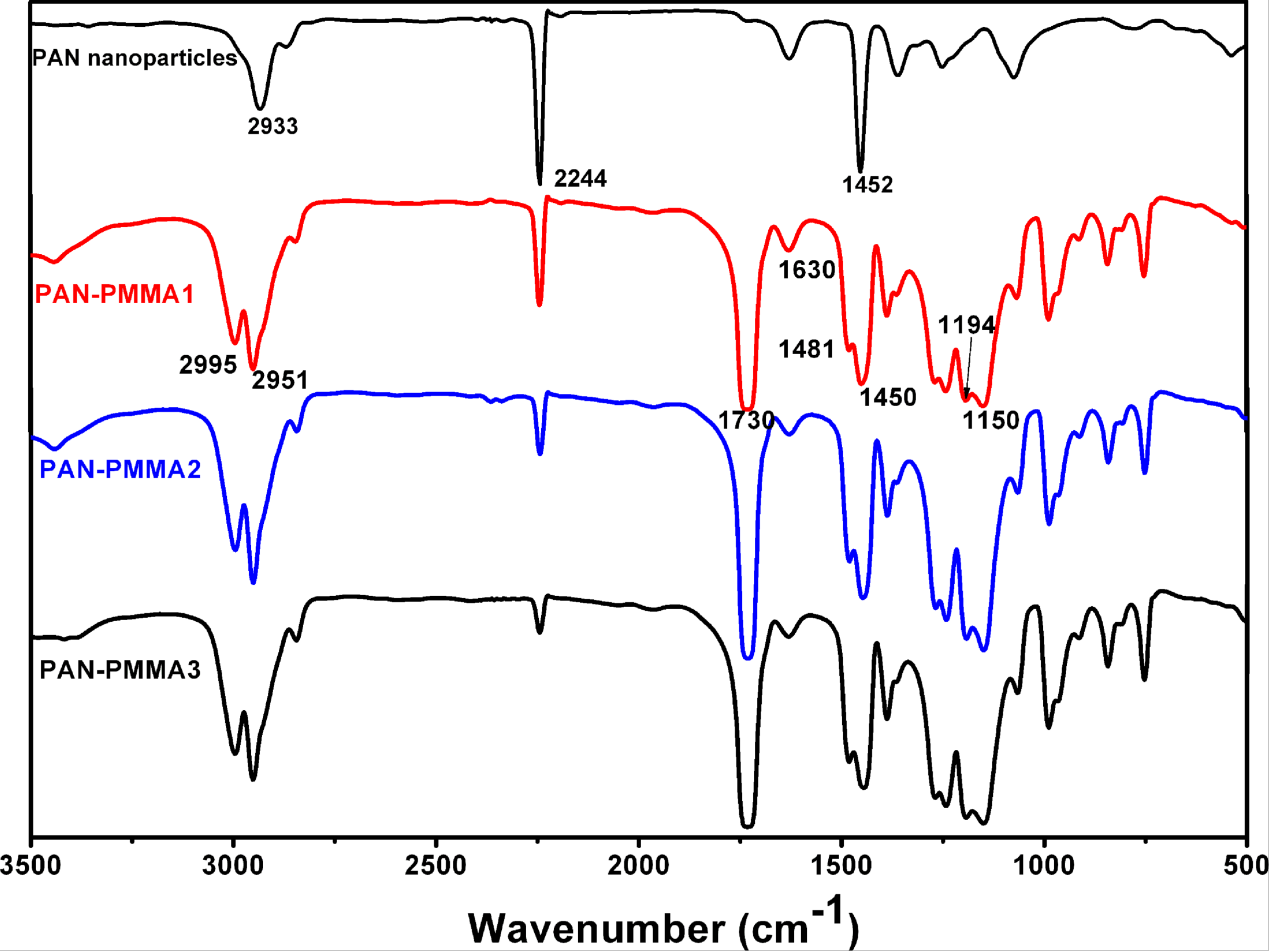
FTIR spectra of PAN nanoparticles and three PAN–PMMA nanoparticles.

Figure 3 displays the FTIR spectra of PAN nanoparticles and three PAN–PMMA nanoparticles. In the spectrum of PAN nanoparticles, the strong adsorption band at 2244 cm^−1^ is assigned to *ν*(C≡N). In the spectra of all the three PAN–PMMA nanoparticles, besides the absorption of *ν*(C≡N) at 2244 cm^−1^, the absorption bands of *ν*_as_(–CH_3_), *ν*_as_(–OCOCH_3_), *ν*(C=O), *δ*_as_(–CH_3_) and splitting *δ*_as_(C–O–C) also appeared at 2995 cm^−1^, 2951cm^−1^, 1730 cm^−1^, 1481 cm^−1^, 1194 cm^−1^ and 1150 cm^−1^, respectively. This reveals the presence of both components, PAN and PMMA, in the three PAN–PMMA nanoparticle samples. Furthermore, as the feeding volume ratio of MMA to AN increased stepwise from 0.8 to 1.6, and to 2.4, the *ν*(C≡N) absorption peak of PAN–PMMA nanoparticles became smaller, and the absorption area ratio of *ν*(C=O) to *ν*(C≡N) increased stepwise from 4.76 to 12.01, and to 20.54. This indicates that the composition of PAN–PMMA nanoparticles can be well controlled by simply adjusting the feeding monomer ratio.

### TEM images

**Figure 4:**
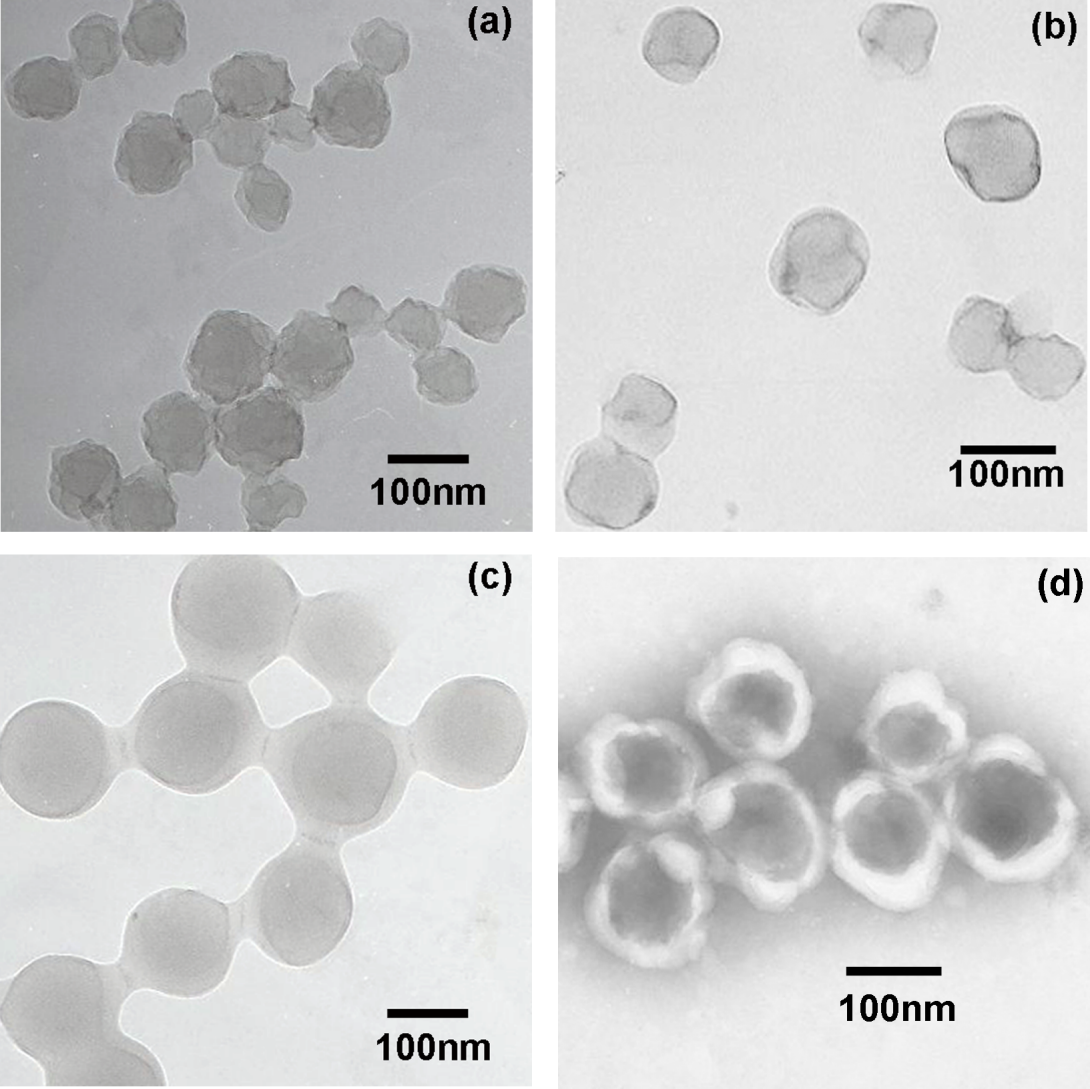
TEM micrographs of various nanoparticles (a) PAN, (b) PAN-PMMA1, (c) PAN-PMMA2 and (d) PAN-PMMA1 treated by 50% NaSCN aqueous solution.

The TEM micrographs of the PAN nanoparticles and the two PAN–PMMA nanoparticles are displayed in Figure 4. As disclosed by Figure 4a, the staining agent phosphate tungsic acid (PTA) molecules could diffuse into and interact with the PAN nanoparticles. Thus, the PAN nanoparticles were well-stained by PTA and are displayed as dark particles. In contrast, PMMA nanoparticles are displayed as white particles with a dark contour (Supporting Information File 1, Figure S3a), indicating PTA molecules could not stain the PMMA nanoparticles [41]. Furthermore, the PMMA particles were almost circular with only one smooth contour line, which is the typical TEM morphology of polymeric latex particles [29]. However, for the PAN particles, several unsmooth contour lines could be discerned, indicating their rough surfaces [39]. A similar morphology was observed in the PAN nanoparticles synthesized by (mini-)emulsion polymerizations [42–43]. Due to the strong dipole between the nitrile groups, the oligomeric PAN chains precipitate and form a nanocrystalline grain structure, leading to the coarse surfaces.

The PAN-PMMA1 nanoparticles synthesized at a MMA/AN FVR of 0.8 are displayed as a core–shell structure composed of dark inner cores with irregular contours and light outer layers with smooth contours (Figure 4b). The presence of smooth outer contours indicates the successful growth of the outer PMMA layer on the surface of PAN seed particles. The distinct irregular contours of the inner cores demonstrate that the outer PMMA layer of PAN-PMMA1 particles is fairly thin. As the FVR increased to 1.6, the PAN-PMMA2 nanoparticles displayed the typical core–shell morphology: thick in the middle and shallow in the periphery (Figure 4c). Meanwhile, not only was the outer contour of the whole particles smooth and spherical, but also the contour of the inner cores became smooth and spherical.

The average diameters of the four particle samples PAN, PAN-PMMA1, PAN-PMMA2 and PAN-PMMA3 obtained by TEM observations were 90 nm, 105 nm, 146 nm and 174 nm, respectively, which are close to those of the DLS results (Supporting Information File 1, Table S3).

The core–shell structure of the PAN–PMMA nanoparticles was also revealed by switching the dispersion medium from water to 50% NaSCN aqueous solution, a selective solvent for PAN. Figure 4d shows the TEM image of the dispersion of PAN-PMMA1 nanoparticles in 50% NaSCN aqueous solution. It can be seen that the PAN-PMMA1 nanoparticles displayed a typical core–shell morphology with dark cores and white shells. After switching the dispersion medium from water to 50% NaSCN aqueous solution, more and more Na^+^ and SCN^−^ ions entered into the inner region of the particles by diffusion. As the concentration of NaSCN in the cores increased gradually, the irregular crystal PAN cores swelled and dissolved and their diameter became larger. Then, the outer PMMA shells were forced to expand accordingly. This phenomenon was also observed in the cavitation of non-covalently-connected micelles composed of carboxyl-ended polybutadiene cores and crosslinked poly(vinyl alcohol) shells in THF/water (9/1, v/v) mixture [44]. It was very possible that the size of PAN chains was too large to diffuse out of the PMMA shell, thus, the dissolved PAN core remained inside the nanoparticles. During the drying of the TEM sample, many NaSCN molecules remained in the PAN cores that could not be removed by the follow-up water washing, thus making them appear dark. The presence of NaSCN molecules enlarged the PAN cores, making the particle diameter (120 ± 13 nm) larger than that of the original untreated ones (105 ± 20 nm).

### SEM images

**Figure 5:**
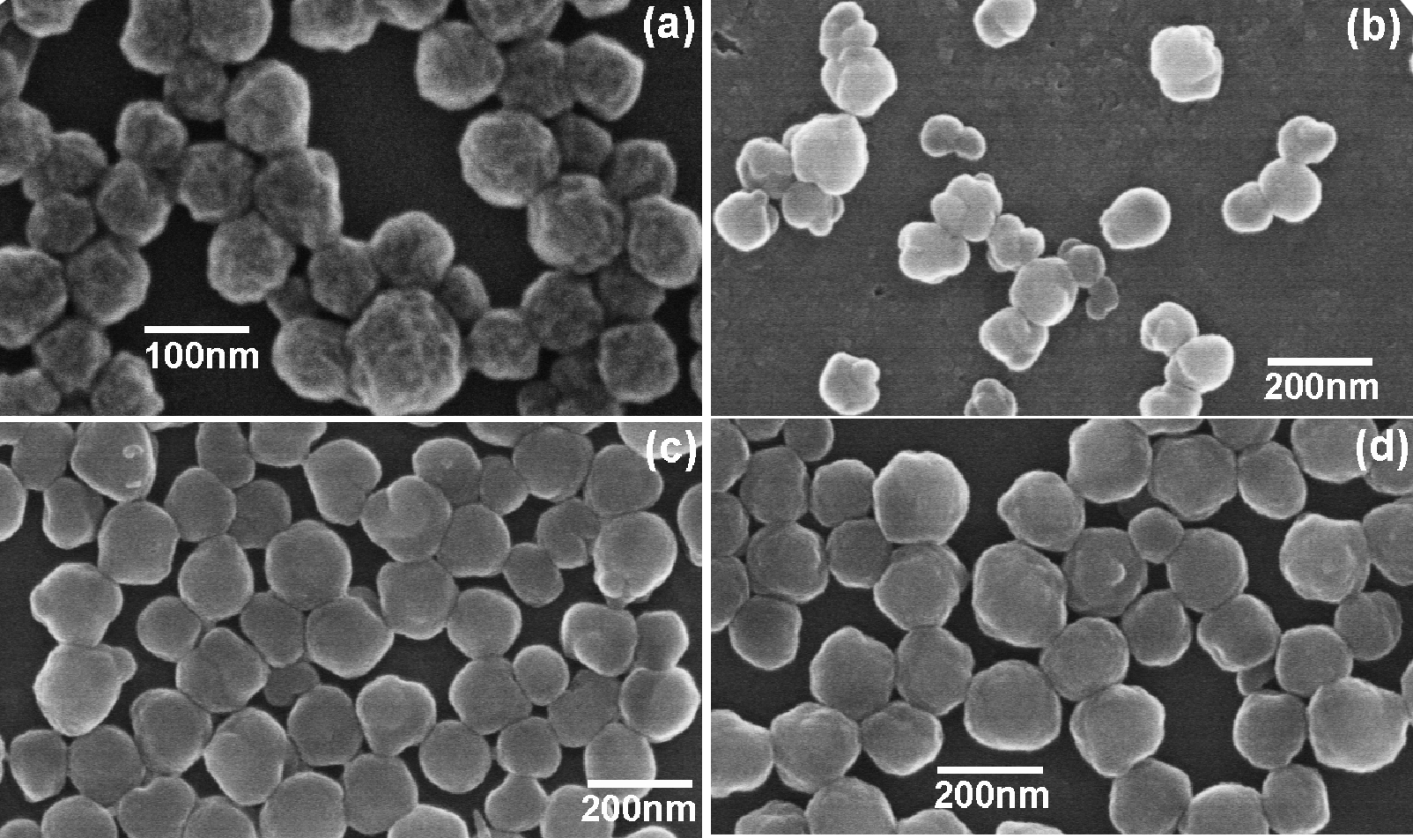
SEM micrographs of various nanoparticles (a) PAN, (b) PAN-PMMA1, (c) PAN-PMMA2 and (d) PAN-PMMA3.

SEM was also used to observe the microstructure of various nanoparticles. As shown in Figure 5a, the original PAN nanoparticles look like “preserved plums” with shriveled surfaces. In contrast, the surfaces of all the three PAN–PMMA nanoparticles are no longer shriveled (Figure 5b−d). At an FVR of 0.8, due to the thin PMMA outer layer, the presence of a coarse inner PAN core leads to large bulges on the surfaces of the PAN-PMMA1 particles (Figure 5b). With increasing FVR, the outer layer became thicker, and the coarse inner PAN cores were buried deep in the outer layer. Thus the PAN–PMMA particles became increasingly close to the smooth spheres. At an FVR of 2.4, only tiny bulges were present on the surfaces of the PAN-PMMA3 particles (Figure 5d). Finally, in terms of the diameter, the morphology revealed by TEM and SEM (Supporting Information File 1, Figure S3) of the PMMA nanoparticles, which are very different from those of the PAN–PMMA nanoparticles, showed that no secondary PMMA nanoparticles were present in both the TEM micrographs and the SEM micrographs of PAN–PMMA nanoparticles.

On the basis of the results of DLS, FTIR, TEM and SEM, it can be concluded that the PAN–PMMA nanoparticles with PAN cores and PMMA shells were successfully synthesized by the two-stage AIBN-initiated semicontinuous emulsion polymerization, although MMA is more hydrophobic than AN. Additionally, the undesirable secondary nucleation is completely inhibited during the AIBN-initiated semicontinuous seed emulsion polymerization. Thus, the PAN–PMMA core–shell structured nanoparticles can be controllably synthesized.

### PAN-based carbon nanospheres

#### Preoxidized product

During the preoxidation process, complicated competitive thermal reactions, including oxidation, cyclization and dehydrogenation, take place. The reactions lead to the formation of a ladder structure, which makes the oxidized PAN infusible during the carbonization treatment and thus increases the carbon yield [45–46]. The X-ray photoelectron spectroscopy result by Vowinkel et al. revealed the formation of pyridine groups after preoxidizing the film composed of SiO_2_@poly(styrene-co-AN) core–shell particles at 240 °C [30].

To optimize the preoxidation temperature of PAN–PMMA nanoparticles, the PAN-PMMA1 nanoparticles were preoxidized at 200 °C, 220 °C, 230 °C, 240 °C and 250 °C. The color of the preoxidized product darkened with the increase of the preoxidation temperature. Dark products were obtained after preoxidation at a temperature of 240 °C or higher. This color change of the preoxidized product was caused by the absorption of light waves by the conjugated structure formed via the cyclization reactions. Furthermore, as the preoxidation temperature increased to 250 °C or higher, dark preoxidized products in hard lumps (instead of powders) were obtained (Supporting Information File 1, Figure S4). This is caused by the softening, flow and agglomeration of the outer nanometer-sized PMMA shell domain during the preoxidation treatment.

**Figure 6:**
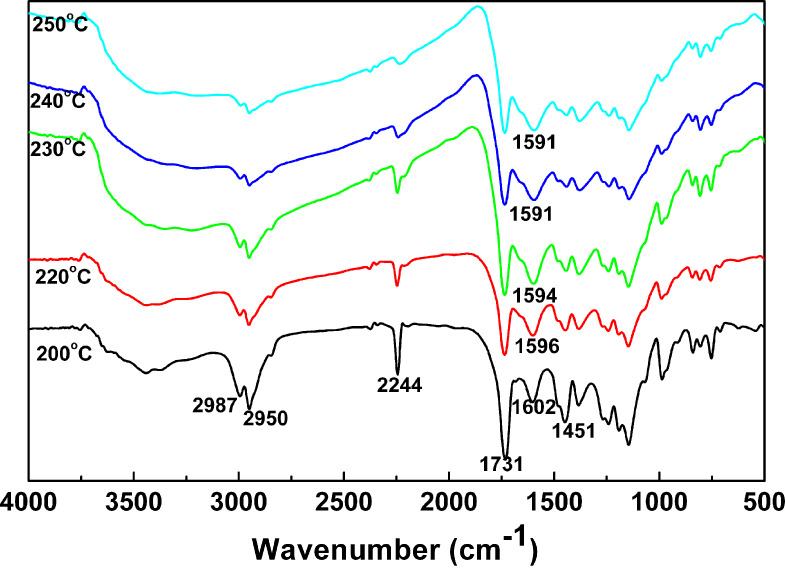
FTIR spectra of the preoxidized products obtained after preoxidizing PAN-PMMA1 nanoparticles at different temperatures.

FTIR was used to study the structure change of PAN-PMMA1 nanoparticles after the preoxidation treatment. By comparing the spectrum of the PAN-PMMA1 nanoparticles (Figure 3) with those of the preoxidized products (Figure 6), it can be seen that, due to the dehydrogenation reaction, the absorptions at 2987 cm^−1^, 2950 cm^−1^ and 1451 cm^−1^ decreased after the preoxidization, and the decrease became larger at higher oxidization temperatures. Furthermore, after the preoxidization, the *v*(C≡N) band became weak, whereas the band around 1630 cm^−1^ intensified and shifted to 1602 cm^−1^. The band continued to shift to lower wavenumbers until 1591 cm^−1^ with an increase in the preoxidization temperature. These changes reflect the contribution of the *v*(–C=N–) band at 1591 cm^−1^ caused by the cyclization reactions [41]. Finally, the *v*(C≡N) band became very weak when the preoxidization temperature reached 250 °C. Thus, to ensure the full cyclization reactions of the PAN chains, the lowest preoxidization temperature of the PAN–PMMA nanoparticles should be 250 °C. At a heating rate of 10 °C/min under nitrogen atmosphere, the temperatures of PMMA nanoparticles with a mass loss of 2% and 5% were 193 °C and 293 °C, respectively (Supporting Information File 1, Figure S5). We chose to preoxidize the PAN–PMMA nanoparticles at 250 °C, considering the limited thermal stability of PMMA outer layer.

#### Carbonized product

Three carbonized products were obtained after the PAN nanoparticles, the PAN-PMMA1 nanoparticles and the PAN-PMMA2 nanoparticles were successively subjected to a preoxidation treatment at 250 °C and a carbonization treatment at 600 °C. The obtained carbonized products were first ultrasonically dispersed in acetone at a concentration of 1.0 g/L. Then, the dispersions were left to stand overnight. The carbonized product of the PAN nanoparticles remained settled at the bottom of the bottle during the ultrasonic treatment (Supporting Information File 1, Figure S6a), indicating the strong adhesion between the carbon nanoparticles. The carbonized product of the PAN-PMMA1 nanoparticles could be completely dispersed in acetone by ultrasonic treatment. However, after storing overnight, most of the carbonized product settled at the bottom of the bottle, and the color of the dispersion faded obviously (Supporting Information File 1, Figure S6b). In contrast, the acetone dispersion of the carbonized product of PAN-PMMA2 nanoparticles (Supporting Information File 1, Figure S6c) still remained stable after overnight storage. These facts suggest that the adhesion between the carbon nanoparticles can be better inhibited when the PMMA outer layer of the precursor is thick enough.

Table 1 lists the elemental content of C, H, N and O in the preoxidized product (POP) and the carbonized products (CP1−CP6) of PAN-PMMA2 nanoparticles. After the preoxidation treatment at 250 °C, the O content of the sample increased substantially. After the further carbonization treatment at 600 °C, the H and O content of the sample decreased while the C and N content increased (POP vs CP1). As the carbonization temperature increased, the C content of the sample increased whereas the N content decreased (CP1–CP4). As compared with the one-carbonization-treatment process, the two-carbonization-treatment process, which combined a low-temperature carbonization and a high-temperature carbonization step, results in product with a higher C content and a higher N content (CP4 vs CP5). After two carbonization treatments at 600 °C and 1000 °C, the C content of the product reached about 85%.

**Table 1:** Elemental content of C, H, N, and O of the preoxidized product (POP) and the carbonized products (CP1–CP6) of PAN-PMMA2 nanoparticles.

Sample	*T* (°C)	C (%)	H (%)	N (%)	O (%)^a^

PAN-PMMA2	–	62.9	7.5	7.9	21.7
POP	250	58.8	6.1	7.6	27.5
CP1	600	61.5	2.4	18.9	17.2
CP2	650	61.8	2.7	17.6	17.9
CP3	700	62.9	2.7	13.3	21.1
CP4	750	65.6	2.4	10.8	21.2
CP5	600–750^b^	68.8	2.8	12.9	15.5
CP6	600–1000^b^	84.9	1.1	4.2	9.8

^a^The O content was obtained by calculation, i.e., O(%) = 100−C−H−N; ^b^CP5 and CP6 were obtained using a two-carbonization-treatment process.

**Figure 7:**
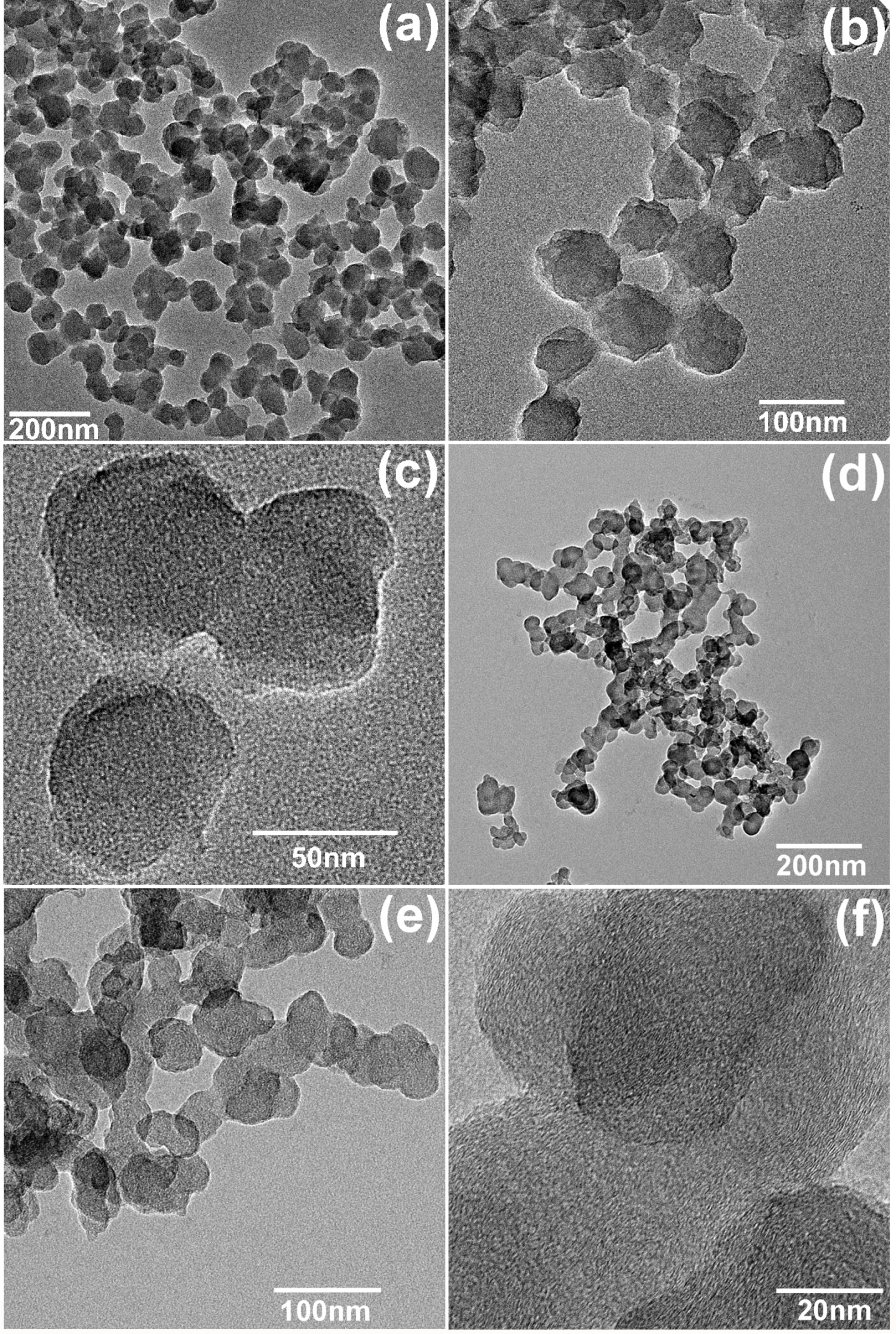
TEM micrographs of the carbonized samples CP1 (a, b, c) and CP6 (d, e, f).

Figure 7 displays the TEM micrographs of the carbonized samples CP1 and CP6 from PAN-PMMA2 nanoparticles after the one-carbonization-treatment at 600 °C and two-carbonization-treatment at 600 °C and 1000 °C, respectively. Single carbon nanoparticles can be clearly discerned in the agglomerates of CP1 and CP6. However, clear adhesion occurred between the particles of CP1 and CP6: the contacting edges of neighboring carbon nanospheres were fused together, and the adhesion was more serious for CP6. The diameter of the particles in the CP1 sample (63–98 nm) was larger than that of CP6 (35–65 nm). This may be caused by the residual of PMMA shell due to the fairly low carbonization temperature (600 °C).

The low viscosity of PMMA fluid formed during the preoxidation treatment enables the agglomeration between the PAN nanoparticles, and thus fails to completely prevent the adhesion between carbon nanospheres after the carbonization treatment. The softening and flow of the PMMA outer layer during the preoxidation treatment can be inhibited by the formation of three-dimensional crosslinked networks. Discrete carbon nanospheres were obtained from PAN-cPMMA2 nanoparticles with divinylbenzene-crosslinked PMMA shells (Supporting Information File 1, Figure S7).

The XRD and Raman spectrum were used to characterize the structure of the carbon nanospheres CP1, CP5 and CP6 (Supporting Information File 1, Figure S8 and Figure S9). In the XRD pattern of the carbon nanospheres, the peaks located at around 24° and 44° are ascribed to (002) planes and (101) planes of graphite carbon, respectively (Supporting Information File 1, Figure S8). The peaks are fairly broad, indicating the obtained carbon nanospheres are amorphous. This is also confirmed by the result of the Raman spectrum. The Raman spectrum (Supporting Information File 1, Figure S9) of carbon nanospheres CP1 displayed two distinct bands at 1345 cm^−1^ and 1588 cm^−1^. The former is called the D-band and is related to the breathing modes of sp^2^ carbon atoms within rings; while the latter is called the G-band and is related to bond stretching of sp^2^ carbon pairs contained in rings or chains. For carbon nanospheres CP5 and CP6, there appeared a new band around 1100 cm^−1^, which is related to C–C sp^3^ vibrations [30]. The Raman spectra of three carbon nanospheres were deconvoluted by Gaussian fitting into four bands, lying around 1100 cm^−1^, 1350 cm^−1^, 1500 cm^−1^, and 1590 cm^−1^. The relative intensity ratio of the D band to G band (*I*_D_/*I*_G_) can be used to characterize the structure ordering of carbon [47–48]. Supporting Information File 1, Table S4 lists the Raman shift, full width at half maximum and intensity of the D band and G band obtained by Gaussian fitting. It can be seen that the *I*_D_/*I*_G_ value of the carbon nanospheres CP1, CP5 and CP6 decreased stepwise, indicating the degree of carbon structure ordering increased with the increase of the carbonization temperature. Additionally, the fairly large *I*_D_/*I*_G_ of CP6 suggests the extent of graphitization of the obtained carbon nanospheres is still very low. This is due to the fairly low carbonization temperature.

**Figure 8:**
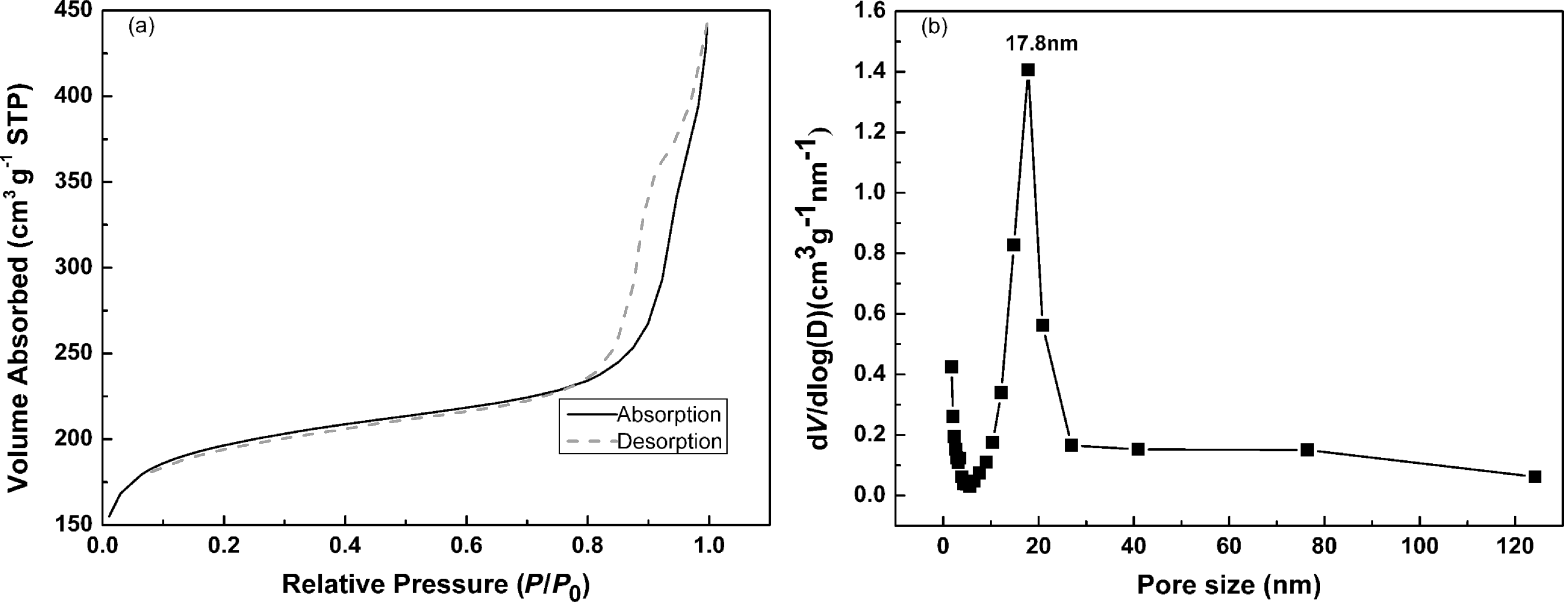
Nitrogen adsorption/desorption isotherm (a) of the carbonized sample CP6 and the corresponding pore size distribution (b) calculated from the desorption branch of nitrogen isotherm using the BJH method.

The pore structure of the carbonized sample CP6 was characterized by nitrogen physisorption. As shown in Figure 8a, CP6 displayed a IV-type adsorption isotherm with H3 hysteresis, indicating the presence of mesopores. This was consistent with the result of the pore size distribution displayed in Figure 8b. The pore size of CP6 ranged from 1.75 nm to 125 nm, with majority of them lying in the range of mesopores. The calculated BET specific surface area of CP6 reached 612.8 m^2^/g, which is considered significant in this family of carbon microspheres and nanospheres [15,26–27,37–38,49].

During the carbonization treatments, cracking reactions occurred in the preoxidized samples [50]. Noncarbon elements including hydrogen, oxygen and nitrogen were removed. Small molecule gases including H_2_O, NH_3_, HCN, CO_2_, N_2_ and alkyl fragments were produced, which caused the formation of many micropores in the PAN-based carbon nanofibers [28]. Here, the presence of an outer PMMA layer, which might be fully decomposed above 600 °C, may impede the timely discharge of the gases from the PAN-based nanoparticles. The formation of large pores, i.e., mesopores may be attributed to the residual gases which remain inside the nanoparticles.

The carbonized sample CP6 was further applied as an adsorbent for removal of methylene blue (MB) (a common dye used as a model for organic pollutants) from water. The absorption of MB on CP6 was very fast; the adsorption capacity and the removal efficiency of MB after 2 h of adsorption reached 190.0 mg/g and 96.1%, respectively. The MB adsorption capacity of CP6 is higher than most of those reported carbon nanomaterials, such as rattle-type magnetic carbon nanospheres (45.15 mg/g) [49], magnetic oxidized multiwalled carbon nanotube- κ-carrageenan-Fe_3_O_4_ nanocomposites (46.36 mg/g) [51], graphene (185.00 mg/g) [52], γ-Fe_2_O_3_ nanocrystal-anchored macro/mesoporous graphene (216.3 mg/g) [53], Fe_3_O_4_-graphene@mesoporous SiO_2_ (178.49 mg/g) [54], manganese-impregnated zinc sulphide nanoparticles deposited on activated carbon (191.57 mg/g) [55] and γ-Fe_2_O_3_ loaded active carbon (195.55 mg/g) [56]. We believe the efficient removal of MB is mainly attributed to the small pore size and the high specific surface area of CP6. This makes the obtained carbon nanospheres excellent adsorbents for the potential removal of organic pollutants from water.

## Conclusion

To summarize, by a combination of emulsion polymerization and pyrolysis, we demonstrated a facile and efficient route to fabricate discrete, PAN-based carbon nanospheres. Core–shell PAN–PMMA nanoparticles at high concentration and low surfactant content were controllably synthesized by a two-stage AIBN-initiated semicontinuous emulsion polymerization method. The thickness of the PMMA outer layer can be easily adjusted by varying the feeding volume ratio of MMA to AN. The adhesion between the carbon nanoparticles, caused by the interparticular cyclization reactions of PAN nanoparticles occurring during the heat treatments, could be efficiently inhibited by a thick PMMA outer layer. Carbon nanospheres with a diameter in the range of 35–65 nm were obtained from the PAN–PMMA2 nanoparticles synthesized at a FVR of 1.6. The obtained carbon nanospheres bear a high BET specific surface area of 612.8 m^2^/g and display a quick and large adsorption of MB. This makes them excellent adsorbents for the removal of organic pollutants from water.

## Supporting Information

Typical UV–vis spectrum of MB aqueous solution and related data for the absorption experiment; Element data of PAN-based nanoparticles; TEM and SEM micrographs of PMMA nanoparticles; Average particle diameter of PAN-based nanoparticles determined by TEM and DLS; Photograph of preoxidized products of PAN-PMMA2 nanoparticles; TG curve of PMMA nanoparticles; Photograph of acetone dispersions of carbonized products after overnight storage; TEM micrographs of carbonized products of shell-crosslinked PAN-cPMMA2 nanoparticles; X-ray diffraction patterns, Raman spectra and related data of the D-band and G-band of carbonized samples.

File 1 Additional experimental details.
