# Supplementary material for: Fabrication of carbon nanospheres by the pyrolysis of polyacrylonitrile–poly(methyl methacrylate) core–shell composite nanoparticles
Source: Beilstein J Nanotechnol. 2017 Sep 11;8:1897–908. doi: 10.3762/bjnano.8.190 (PMC5629418; doi:10.3762/bjnano.8.190)
Supplement: File 1 — Additional experimental details. [file Beilstein_J_Nanotechnol-08-1897-s001.pdf]

Supporting Information

for

Fabrication of carbon nanospheres by the pyrolysis of polyacrylonitrile–poly(methyl methacrylate) core–shell composite nanoparticles

Dafu Wei[1], Youwei Zhang[*2] and Jinping Fu[2]

Address: [1]Key Laboratory for Ultrafine Materials of Ministry of Education, School of Materials Science and Engineering, East China University of Science and Technology, Shanghai 200237, China, and [2]State Key Laboratory for Modification of Chemical Fibers and Polymer Materials, College of Materials Science and Engineering, Donghua University, Shanghai 201620, China

Email: Youwei Zhang* - zhyw@dhu.edu.cn

* Corresponding author

Additional Experimental Details

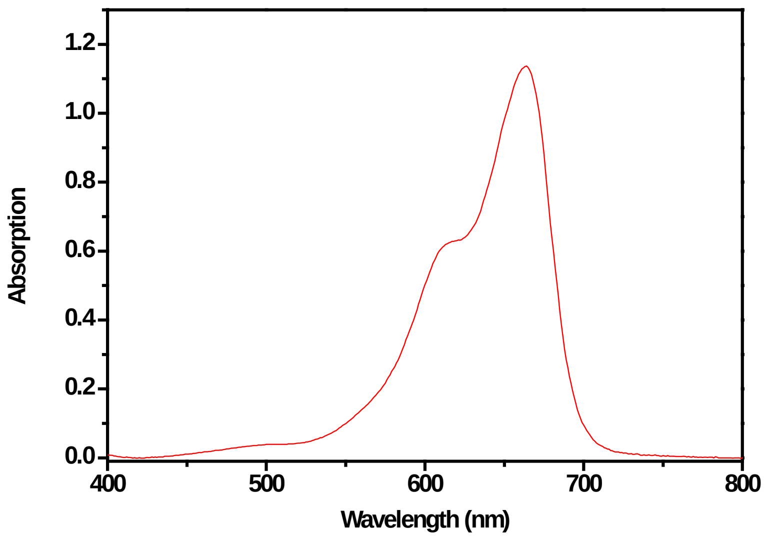

Figure S1: UV–vis spectrum of MB aqueous solution.

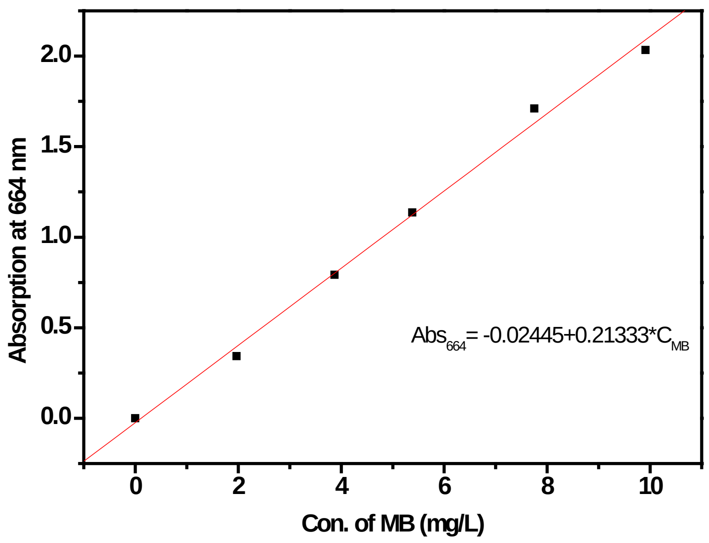

Figure S2: Calibration curve of MB aqueous solutions.

Table S1: Absorption of a series of MB aqueous solutions with known concentrations and supernatant of the MB/carbon-nanospheres/water mixture at λ = 664 nm.

Sample	C of MB (mg/L)	Absorption [a]
MB aqueous solution	1.97	0.3425
	3.87	0.7918
	5.38	1.1367
	7.75	1.7106
	9.91	2.0327
Supernatant obtained by centrifuging the MB/carbon-nanospheres/water mixture after the adsorption experiment	2.14[b]	0.4313

[a]average value for three measurements; [b]calculated according to the calibration equation displayed in Figure S2.

Table S2: Content of elements C, H and N of PAN seed nanoparticles and PAN-PMMA composite nanoparticles.

Sample	*FVR* (MMA/AN,v/v)	C(%)	H(%)	N(%)	*WR*[a]
PAN latex	-	67.14	5.749	25.72	-
PAN-PMMA1	0.8	63.32	7.047	12.04	1.136
PAN-PMMA2	1.6	62.23	7.456	7.942	2.238
PAN-PMMA3	2.4	61.71	7.603	6.154	3.179

[a]The weight ratios (*WR*) of PMMA to PAN of the PAN-PMMA nanoparticles were estimated from the N content of the PAN seed nanoparticles and the PAN-PMMA nanoparticles determined by elemental analysis.

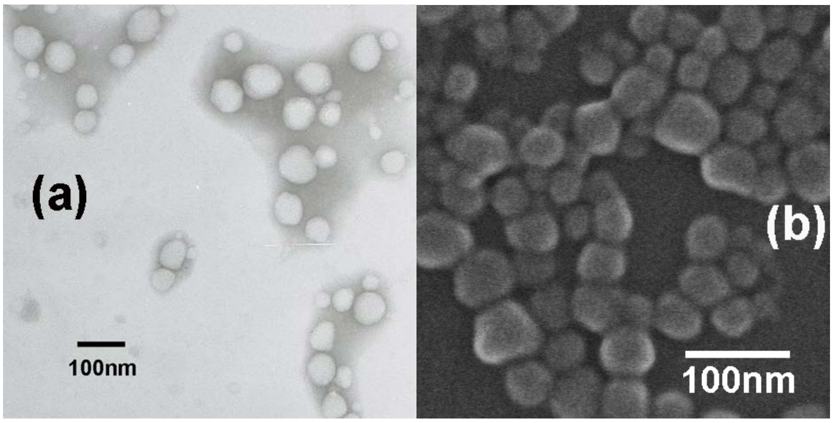

Figure S3: TEM (a) and SEM (b) micrographs of PMMA nanoparticles.

Table S3: Average particle diameters by TEM (D_{TEM}) and DLS ($<D_h>$) measurements of PAN seed nanoparticles and PAN-PMMA composite nanoparticles.

Sample	D_{TEM} (nm)	$<D_h>$ (nm)[a]
PAN	90±16	97±1
PAN-PMMA1	105±20	122±3
PAN-PMMA2	146±12	154±2
PAN-PMMA3	174±10	178±6
PAN-PMMA1 treated by 50% NaSCN aqueous solution	120±13	

[a]The average value and standard error for the z-average hydrodynamic diameters of three measurements.

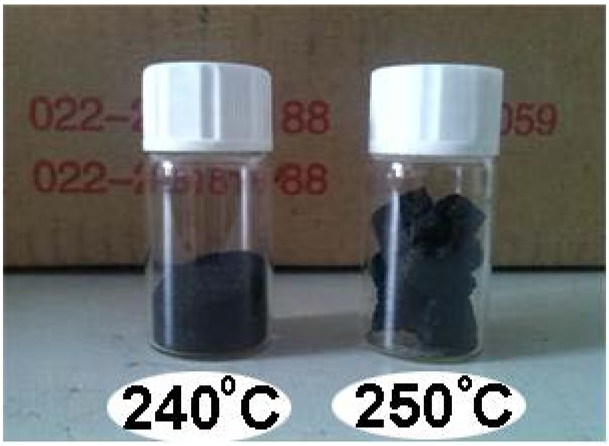

Figure S4: Pre-oxidized products obtained after pre-oxidizing PAN-PMMA2 nanoparticles at 240 °C and 250 °C.

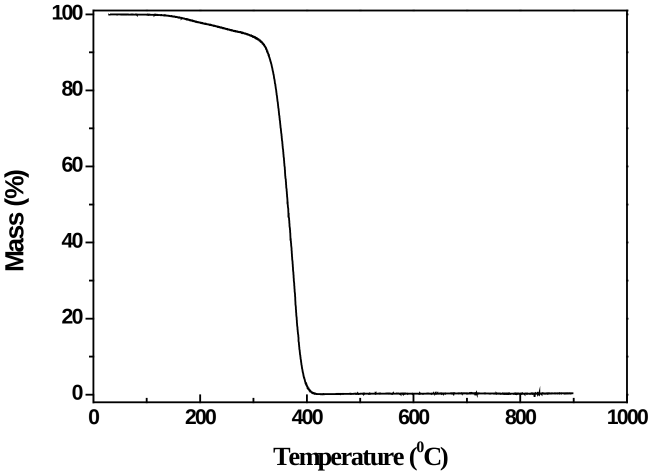

Figure S5: Thermogravimetric (TG) curve of PMMA nanoparticles at a heating rate of 10 °C/min under nitrogen protection.

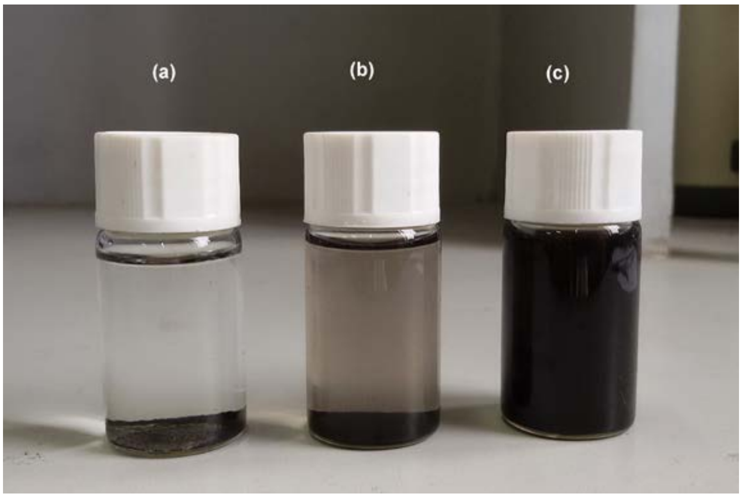

Figure S6: The appearance of acetone dispersions of three carbonized products (the ultrasonication was used for dispersing various carbonized products in acetone) of PAN nanoparticles (a), PAN-PMMA1 nanoparticles (b) and PAN-PMMA2 nanoparticles (c) after overnight storage.

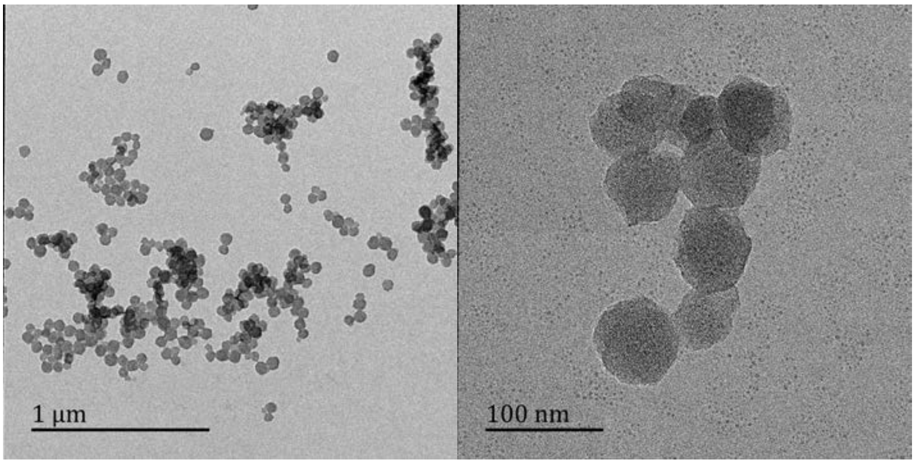

Figure S7: TEM images of carbonized products obtained after successively subjecting the PAN-cPMMA2 nanoparticles with DVB-crosslinked PMMA shell to pre-oxidization treatment at 250 °C and two carbonization treatments at 600 °C and 1000 °C.

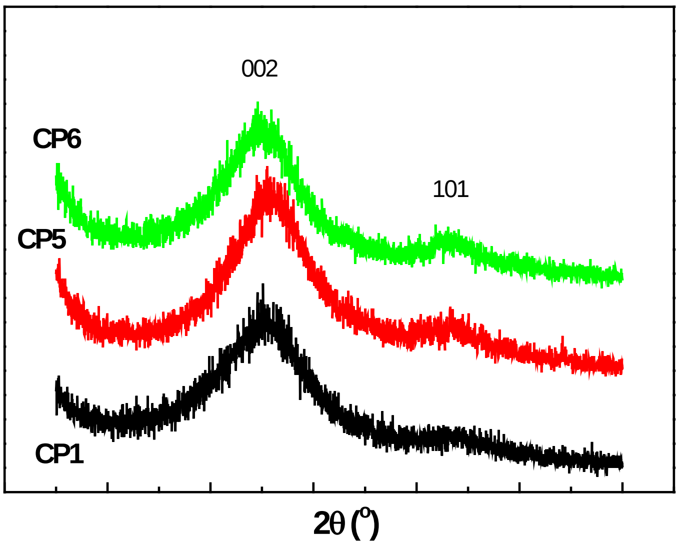

Figure S8: X-ray diffraction patterns of carbonized samples CP1, CP5 and CP6.

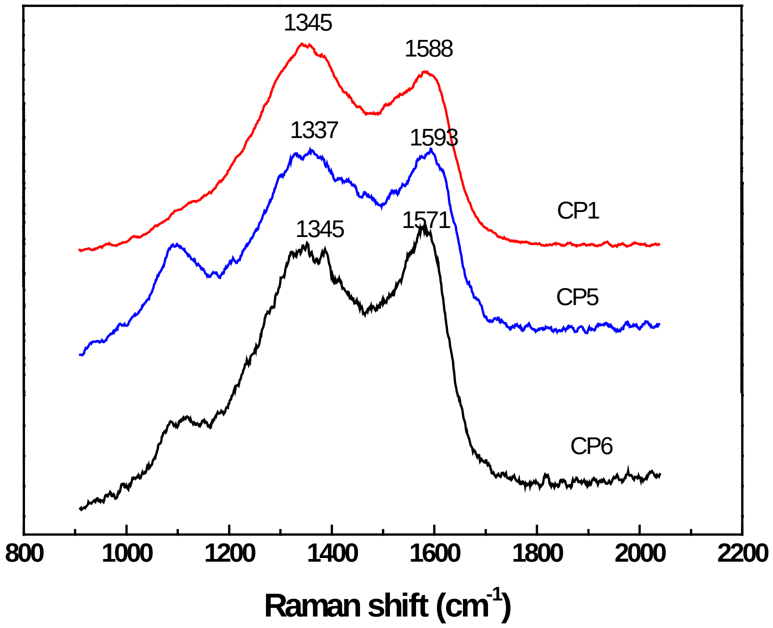

Figure S9: Raman spectra of carbonized samples CP1, CP5 and CP6.

Table S4: Raman shift, full width at half maximum and intensity of D band and G band of three carbon nanospheres obtained by Gauss fitting.

Sample	D Band			G Band			I_D/I_G
	Raman shift (cm^{-1})	FWHM[a] (cm^{-1})	Intensity (a.u.)	Raman shift ($cm^{-1)}$	FWHM[a] (cm^{-1})	Intensity (a.u.)	
CP1	1352.9	290	154	1578.6	133	51.9	2.97
CP5	1367.3	274	155	1591.2	121	56.6	2.74
CP6	1362.4	272	114	1580.4	116	43.7	2.61

[a]FWHM - full width at half maximum.
